# Student Apprenticeship to General Practitioners

**Published:** 1956-10

**Authors:** W. M. Capper

**Affiliations:** Clinical Dean, University of Bristol


### STUDENT APPRENTICESHIP TO GENERAL PRACTITIONERS

BY

W. M. CAPPER, F.R.C.S.

*Clinical Dean, University of Bristol*

Since 1953 there have been two major changes in the clinical training of students in this University. One is in the first three months and the second in the last three months of the course. The first is that for a week during the pre-clinical course every student is attached to a ward to do the ordinary work of a nurse. This means that every student has first-hand experience in the general nursing care of the patient in the wards. The other major change has been that an opportunity is now given to every student during the last three months of his training to be attached to a general practitioner. This was started in 1954 and has been carried out every year since.

In 1954, 36 students took part in the scheme; in 1955 there were 30, and in 1956, 28. Students were attached to 6 country practices and 18 town practices and the list of practitioners asked to participate has been drawn up every year by a committee appointed for the purpose. Briefly the scheme consists of attaching one or two students to a general practitioner in or near Bristol for a period of a fortnight during the months of March and April before the finals in June. The student usually reports to the practitioner for the morning surgery and goes with him on his rounds during the day, seeing such cases as the practitioner decides and attending the evening surgery. He is also on call for any night work. In most cases he attends the doctor throughout the day and has an opportunity of discussing the patients with him and of seeing the general run of practice. In some cases the student lives with the doctor and in others he finds his own lodgings close to the practitioner's surgery.

As far as possible students are set free from any attendances at the University during this time and are able to attend the practitioner throughout the day.

### STUDENTS' APPRECIATION

From the point of view of the student the scheme has met with very considerable appreciation; almost without exception they report that they have thoroughly enjoyed the experience and have enlarged their knowledge considerably. They have been most hospitably entertained by the practitioners. Many students have stated how valuable it has been in getting experience in the doctor-patient relationship, the general approach to patients and the treatment of the common ailments.

### PRACTITIONERS' REACTIONS

The general practitioners for the most part seem to be quite impressed with the knowledge, ability, and keenness of the students. At the end of their time the practitioners send in a report of the students' work which is filed with the students' records.

The reaction to the scheme by practitioners has been variable.

One writes:

"I found the experience most stimulating and interesting. My patients without exception welcomed us. Many of the chronic sick and the neurotics delighted in displaying their troubles to a new doctor. . . . I would be grateful to participate on any future occasion."

Another writes:

"Frankly I doubt if the scheme run as at present is worthwhile. These students come too near the final M.B. to get any benefit at all. Manifestly we cannot show them much but if they are to have not only the looming final but minor day-to-day tutorials to worry about, I think they would be better not to come at all. In my own two examples this year, in hardly any case did either student see the patient twice—the exception being a maternity case we visited a few times. Such an impression of general practice is ridiculous—the whole point is we are dealing with people we see often. I think they should come for at least two weeks. and have no other duties at all during the time they are with us."

And another:

"Both students were most keen on the work, neither missed a single item of surgery or visiting work whilst here; each hung to my tail the whole time. I found both very knowledgeable and most helpful whenever I sought advice as to the kind of treatment this or that case here would receive if treated in hospital. My patients found —— the more forthcoming and sympathetic but all seemed to have liked both of them. I had asked for no opinion at all but have had it given freely at my subsequent interviews with many of them. My opinion is that they are extremely keen young folk, already well versed in general medical knowledge, and both should make good doctors. Both seemed, in quite different ways, to be sound young folk and I feel that if they are a fair sample of the kind of people the University are turning into doctors there need be no anxiety for the future class of doctors."

A fourth makes a suggestion:

"The scheme at present is that a medical student in his final year is apprenticed to a general practitioner for a period of two weeks. This I suggest is far too late in his career. By that time he is already conditioned by several years of hospital work and regards a 'patient' from the hospital angle which is quite different from seeing them in a G.P.'s surgery or in the background of their own homes. Also, this is rather an anxious time for him because he is far too preoccupied in mind about his forthcoming examinations to derive the full benefit from his experience. I feel it would be much better to learn about general practice at the beginning of his clinical studies. As he signs on for his various clinics he would at the same time be apprenticed to a G.P. for the remainder of his medical training. The details of this apprenticeship would provide a fruitful subject for discussion."

The general impression from many letters from practitioners is that they welcomed the scheme but thought that it was rather late in the career of the students to see general practice; also that the students should be absolutely free from all University commitments during the fortnight of apprenticeship.

### UNIVERSITY'S GRATITUDE

From the University point of view the scheme has been satisfactory. It is of course, impossible in the present medical curriculum to free students completely from all University commitments. Indeed, the present curriculum is so heavily loaded with so many and so diverse details that it is difficult to fit in anything extra at all.

There is no doubt that the students have gained by this scheme and we should like to thank all those practitioners who have taken part and who have given freely of their time and energy, and often hospitality, to enable the students to see the inner side of general practice. Without exception there has been whole-hearted co-operation in the scheme, and we are extremely grateful for all that has been done. Whether future changes will be possible along the lines suggested, is not yet determined.

